# Impact of pre-existing depression and food insecurity on the trajectory of depressive symptomatology during the COVID-19 pandemic outbreak in South Africa: A panel analysis of nationally representative South African data

**DOI:** 10.1007/s12571-024-01448-x

**Published:** 2024-07-01

**Authors:** Philile Dladla-Jaca, Busisiwe P. Ncama, Yoshan Moodley, Nafiisa Sobratee-Fajurally, Rashieda Davids, Mjabuliseni Simon C. Ngidi, Catherine Sutherland, Muthulisi Siwela, Tafadzwanashe Mabhaudhi, Albert T. Modi, Rob Slotow, Jonathan K. Burns, Andrew Tomita

**Affiliations:** 1 Department of Public Health Medicine, School of Nursing and Public Health, University of KwaZulu-Natal, Durban, South Africa; 2 Department of Global Health, Faculty of Medicine and Health Sciences, Stellenbosch University, Cape Town, South Africa; 3 School of Life Sciences, University of KwaZulu-Natal, Pietermaritzburg, South Africa; 4 School of Agricultural, Earth and Environmental Sciences, University of KwaZulu-Natal, Pietermaritzburg, South Africa; 5 Centre for Transformative Agricultural and Food Systems, School of Agricultural, Earth & Environmental Science, University of KwaZulu-Natal, Pietermaritzburg, South Africa; 6 African Centre for Food Security, School of Agricultural, Earth and Environmental Sciences, University of KwaZulu-Natal, Pietermaritzburg, South Africa; 7 Department of Agricultural Extension and Rural Resource Management, School of AgriculturalEarth and Environmental Sciences, University of KwaZulu-Natal, Durban, South Africa; 8 School of Built Environment and Development Studies, University of KwaZulu Natal, Durban, South Africa; 9 Centre On Climate Change and Planetary Health, London School of Hygiene and Tropical Medicine, London, United Kingdom; 10 Oppenheimer Fellow in Functional Biodiversity, Centre for Functional Biodiversity, School of Life Sciences, University of KwaZulu-Natal, Pietermaritzburg, South Africa; 11 Department of Genetics, Evolution and Environment, University College, London, United Kingdom; 12 Faculty of Health and Life Sciences, University of Exeter, Exeter, UK; 13 Department of Psychiatry, School of Clinical Medicine, University of KwaZulu-Natal, Durban, South Africa; 14 Centre for Rural Health, School of Nursing and Public Health, University of KwaZulu-Natal, Durban, South Africa; 15 KwaZulu-Natal Research Innovation and Sequencing Platform (KRISP), University of KwaZulu-Natal, Durban, South Africa

**Keywords:** Food security, Covid-19, Depression, South Africa

## Abstract

We investigated the trajectory of depressive symptoms (“depression”) from the start of the COVID-19 pandemic in South Africa (March 2020) until 2021, between individuals with and without pre-pandemic depression, specifically regarding the role of food security. Our investigation used publicly available panel data (N = 6,930) from the South African National Income Dynamics Study Coronavirus Rapid Mobile Survey (SA-NIDS-CRAM from 2020–2021) on those who had also participated in the pre-pandemic South African National Income Dynamics Study (SA-NIDS, 2017) depression interview. We investigated trends in depressive symptomatology (based on a 2-item Patient Health Questionnaire) at SA-NIDS-CRAM Wave 2 (July 2020), Wave 3 (February 2021) and Wave 5 (May 2021). Generalized estimating equations (GEE) with post-estimation linear combinations of estimators were fitted to investigate the roles of pre-pandemic depression (based on 2017 SA-NIDS data) and food insecurity during the pandemic on depressive symptomatology. During the pandemic, the highest levels of depression were observed consistently among those with pre-pandemic depression and food insecurity; and were lowest among those without pre-pandemic depression and food security. Depressive symptomatology rose in nearly equal magnitude during the early phases of the pandemic in two groups: those without pre-pandemic depression but food insecure during the pandemic; as well as those with pre-pandemic depression but food secure during the pandemic. However, this dynamic changed later in the pandemic, when higher depressive symptomatology was observed in the group with both pre-pandemic depression and food insecurity, widening the gap between them from Wave 3 (adj β = 0.63, p < 0.01) to Wave 5 (adj β = 0.79, p < 0.01). Our results highlight the importance of addressing both population mental health and food insecurity, particularly at the early stages of a crisis/disaster. As we showed that mental health impact is linked to food insecurity during a pandemic, strengthening social protection measures, especially around food and nutrition, would help build resilience to crises in the long term.

## Introduction

More than 280 million people, or 3.8% of the world's population, were reported to suffer from depression globally in 2019 (Institute of Health Metrics and Evaluation, 2024). As a leading cause of years lived with disability globally (Global Burden of Diseases 2017 Disease and Injury Incidence and Prevalence Collaborators, 2017), depression is increasingly being recognized as a costly burden (Evans-Lacko & Knapp, 2016) for resource-limited nations pursuing economic development aimed at reducing poverty. According to the Global Burden of Disease study (Liu et al., 2020), there has been a 49.9% estimated increase between 1990 and 2017 worldwide in the incidence of major depressive disorder (depression that is more persistent and potentially severe in symptomatology). Considered one of the most unequal countries in the world (Sulla et al., 2022), approximately one in every 10 adults (9.8%) experience a major depressive disorder in their lifetime in South Africa (Herman et al., 2009). However, due to a lack of capacity and resources in the public health systems (Marais & Petersen, 2015), the treatment gap for mental disorders is estimated to be as high as 92% (Docrat et al., 2019), making it difficult for most individuals to access care for depression.

The COVID-19 pandemic, with a reported 102,395 COVID-19-related fatalities between January 2020 and November 2022 in South Africa (World Health Organization, 2024), placed an additional strain on the under-resourced and over-burdened health system, and disrupted access to healthcare for chronic conditions (Health Systems Trust, 2021). Disasters, including COVID-19, which was declared a national State of Disaster in South Africa (Republic of South Africa, 2020), are defined as unforeseen events that cause great damage, destruction and human suffering, often due to nature or man-made causes (Republic of South Africa, 2003). Section 1(b) of Disaster Management Act No. 57 of 2002 in South Africa further defines disaster as being of a magnitude that exceeds the ability of those affected to cope (Republic of South Africa, 2003). The definition implies that disasters can be emotionally overwhelming experiences for individuals who are socio-economically vulnerable due to limited resources/support to cope with such devastating and stressful events (Hallegatte et al., 2020; Tomita et al., 2022; Troup et al., 2021). Furthermore, disasters may also place additional emotional burdens on individuals facing pre-existing mental health challenges, due to their lack of resources to access treatment. 

Several recent studies point to an alarming rise of depression due to disaster, such as COVID-19 (Bueno-Notivol et al., 2021; Lee et al., 2021; Necho et al., 2021; Kim et al., 2022), including the social inequality in the burden of the pandemic in South Africa (Posel et al., 2021; Posel, 2021). Studies report job-loss (Posel et al., 2021), and social isolation (Posel, 2021) during the COVID-19 lockdown that have contributed to significant depression challenges in South Africa. More worrying is the socio-economic consequence of the pandemic, with individuals not being able to meet basic human needs, such as food security (van der Berg et al., 2022), due to income loss as a result of job losses or work stoppages due to lockdowns in South Africa (Arndt et al., 2020). It is estimated that as many as one quarter of the population in South Africa faced moderate to severe food insecurity during the pandemic compared to 17.3% in 2019 (Statistics South Africa, 2020a). Given the large mental health treatment gap (Docrat et al., 2019) that existed even before the pandemic, an important question is how individuals with (and without) pre-existing depression fared during the (early onset of) pandemic that caused significant socio-economic havoc in South Africa. Our cohort study investigated the trajectory of depressive symptoms from the start of the COVID-19 State of Disaster declaration in South Africa (March 2020) until May 2021, between individuals with and without pre-existing depressive symptoms (i.e. pre-pandemic depression), specifically regarding the role of food security. We posit, based on various social theory, including social causation and social drift (Dohrenwend et al., 1992; Hudson, 2005; Lund et al., 2011; Reiss et al., 2013; Lund and Cois 2018) [explained later], that individuals with pre-pandemic depressive who experienced food insecurity during the pandemic had the highest level of depressive symptomatology, due to a lack of resources combined with mental illness. Given the growing concern regarding disasters (especially in relation to climate change), this current work investigated the roles of pre-pandemic depressive symptoms and food insecurity on depressive symptomatology, to potentially guide disaster planning at a national level.

## Methods

### Study overview

Our investigation involved the publicly available secondary cohort data analyses from the South African National Income Dynamics Coronavirus Rapid Mobile Survey (SA-NIDS-CRAM during 2020–2021), this being a continuation of the South African National Income Dynamics Survey (SA-NIDS), which was undertaken during 2008–2017. Our target sample consisted of the participants (N = 6,930) from SA-NIDS-CRAM who were also part of the SA-NIDS depression interview in 2017. Both the SA-NIDS and the SA-NIDS-CRAM are designed to monitor the socio-economic and health state of South Africa at a population level over time, based on a panel study design. The SA-NIDS interviews were undertaken in five waves (2008, 2010, 2012, 2014 and 2017), with two of the five SA-NIDS-CRAM interviews being undertaken during 2020 (May and Sept) and three in 2021 (February, May and July). Both the SA-NIDS and the SA-NIDS-CRAM are considered nationally representative studies, with sample participants drawn using a stratified sampling design (but with “batch sampling” for the SA-NIDS-CRAM). For the SA-NIDS, a stratified, two-stage cluster sample design was employed, and data was collected based on a face-to-face method. For the SA-NIDS-CRAM, data were collected with Computer Assisted Telephone Interviewing (CATI) due to COVID restrictions. Further details about SA-NIDS (Southern Africa Labour & Development Research Unit, 2018) and SA-NIDS CRAM (Southern Africa Labour & Development Research Unit, 2020) can be found in previously published reports. Ethical clearance for this cohort study was obtained from the University of KwaZulu-Natal Biomedical Research Ethics Committee (BREC/00003286/2021).

### Primary outcome

The primary outcome of the cohort study is depressive symptomatology, which was measured using the 2-item Patient Health Questionnaire (Kroenke et al., 2003). As a valid instrument for use in time-constrained settings such as South Africa (Bhana et al., 2015), the outcome data are only available in the SA-NIDS-CRAM for Waves 2 (September 2020), 3 (February 2021) and 5 (May 2021). Each item in the Patient Health Questionnaire (PHQ-2) is based on four possible responses in a Likert format, from 0 = not all, to 3 = nearly every day. A total PHQ-2 score (i.e. depressive symptomatology level), based on two items, can range from 0–6, with a higher total score indicating a greater level of depressive symptomatology. Importantly, this variable measures the severity of depressive symptoms, not a diagnostic entity. For ease of reading, we use the term “depression” in this paper in place of the term “depressive symptoms” and reiterate this is not equivalent to a diagnosis of depressive disorder.

### Exposures

The two exposure variables of interest were pre-pandemic history of significant depression (hereafter labelled as pre-pandemic depression status) and household food security during the pandemic. History of pre-pandemic depression was based on the 10-item abridged version of the Center for Epidemiologic Studies Depression Scale (CES-D) data (Andresen et al., 1994), which was available in the Adult Questionnaire from SA-NIDS Wave 5 [2017, final pre-pandemic SA-NIDS study]. The CES-D captures depression-associated symptoms during the past week based on self-report and is a commonly used psychometric valid/reliable instrument. Similar to the PHQ-2, the CES-D is based on four possible responses in a Likert format, from 0 = rarely/none of the time to 3 = almost/all of the time. As a valid instrument (Andresen et al., 1994; Cole et al., 2004) for the South African population (Baron et al., 2017), we classified a cumulative score of 10 or greater in the 10-item CES-D composite score as a cut-off to represent significant depressive symptoms, this being consistent with a previous study (Andresen et al., 1994). As per our comment above, we use the term “depression” to indicate significant depressive symptoms (i.e. above the cut-off). Regarding household food security during the pandemic, data were available for Waves 2, 3 and 5 of the NIDS-CRAM study, where participants were asked whether anyone in their household had gone hungry in the last seven days due to insufficient food.

### Analysis

First, the socio-demographic and clinical variables (including the prevalence of pre-pandemic depression) at first entry/assessment in the SA-NIDS CRAM were summarized using descriptive analysis. Second, we investigated the roles that pre-pandemic depression and food security status had on depressive symptomatology during the pandemic by fitting a generalized estimating equation (GEE), using modelling with an exchangeable correlation structure, to account for the correlation of responses within participants’ repeated measures in the panel data (Zeger et al., 1988). Each regression model was adjusted for the socio-demographic (e.g. gender, age, race, employment status, food insecurity) and clinical variables available (history of comorbidity and pre-pandemic depression status). The regression analyses were also adjusted by the panel weight provided by the SA-NIDS CRAM study, these being the SA-NIDS Wave 5 post-stratified weights adjusted for the probability of selection into SA-NIDS-CRAM and subsequently adjusted for non-response (Ingle et al., 2021). Due to the difficulty of interpreting the interaction variables in the regression, we applied post-estimation linear combinations of estimators (lincom) (StataCorp, 2024) to compare significant group differences in the depressive symptomology levels by time point, pre-pandemic depression status and food security status during the pandemic. For clinical context, we also report effect size (ES). For the difference in the mean PHQ-2 score at two assessment periods, ES size is calculated by dividing it (i.e. period difference in the mean PHQ-2 score) by the standard deviation (SD) obtained at baseline (Lowe et al., 2005). As for the ES of the group differences in the PHQ-2 score at a time period, ES size is calculated by dividing it (i.e. group differences in the PHQ-2 score) by SD of the entire sample at the time period consistent with past PHQ-2 studies (Kroenke et al., 2003) Although there is no universal rule of thumb, we interpreted ES being small (≥ 0.2), medium (≥ 0.5) and large (≥ 0.8) (Cohen, 1992). STATA 17 (StataCorp, 2021) was used to analyse the data (Table 1).

**Table 1:** Baseline socio-demographic and clinical cohort profile at first enrolment

Variables	Categories	Overall (N = 6,930)	
		n	**%**
Gender:	Male	2,621	46.0
	Female	4,309	54.0
Age categories:	15–19	284	4.3
	20–24	850	12.2
	25–29	784	13.3
	30–34	1,010	12.6
	35–64	3,279	48.5
	65 +	722	9.1
Race:	African/Black	5,992	80.6
	Coloured	583	9.2
	Asian/Indian	60	1.9
	White	295	8.4
Employment status:	Not employed	4,049	55.5
	Employed	2,809	44.5
History of comorbidity:	Yes	1,317	19.1
	No	4,584	80.9
Food Insecurity:	Yes	1,411	16.7
	No	5,482	83.3
Depression:	Yes	1,589	23.5
	No	5,341	76.5
History of depression (prior to enrolment):	Yes	1,576	21.3
	No	5,354	78.7

When the sample is limited to NIDS-CRAM Wave 2 sample, the depression risk and history of depression was 24% and 21% respectively which matches the work by Oyenubi and Kollamparambil (2020). History of comorbidity based on SA-NIDS CRAM Wave 1 data due to availability. The history of comorbidity refers to health conditions which include HIV, TB, lung condition, heart condition or diabetes as one-item question in the SA-NIDS CRAM Wave 1 questionnaire

## Results

### Socio-demographic and clinical cohort profile

The study consisted of 6,930 participants who were entered into the SA-NIDS CRAM study and for whom information was available on their pre-pandemic depression status (in 2017 from SA-NIDS). The sample characteristics of participants were female (62.2%) and Black African (86.5%), with 9.1% being ages 65 and above, which closely resembles South Africa’s 2020 Mid-year population estimates (Statistics South Africa, 2020b). Approximately 21.3% had pre-existing depression.

### Depressive symptomatology levels in individuals with and without a history of prior depression

**Fig. 1:**
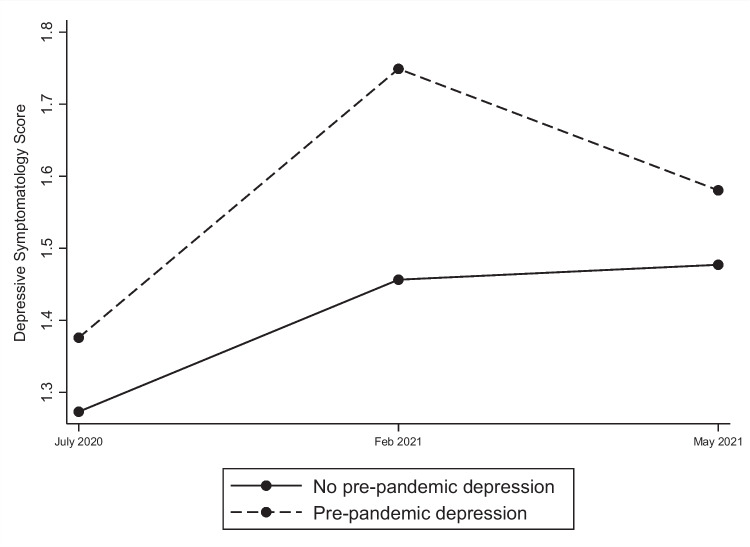
Depressive symptomatology levels in individuals with and without pre-pandemic depression

The trends in depressive symptomatology in individuals with and without a history of prior depression for Waves 2, 3 and 5 during the pandemic are indicated in Fig. 1. Two important findings emerged, the first being that depression levels did not differ significantly between individuals with and without pre-pandemic depression at entry (Wave 2: July 2020). However, at Wave 3 (Feb 2021), we detected significantly greater depression levels in the pre-pandemic depression group than in those without (lincom results: adj β = 0.29, p < 0.01, ES = 0.17). By Wave 5 (May 2021); there was no significant difference between these groups.

Second, we found that depression levels were significantly higher in Wave 3 than in Wave 2 in both those with pre-pandemic depression (lincom results: adj β = 0.37, p < 0.01, ES = 0.22) and without (lincom results: adj β = 0.18, p < 0.01, ES = 0.11). By Wave 5, we detected higher depression levels among those without pre-pandemic depression (lincom results: adj β = 0.20, p < 0.01, ES = 0.13), but not among those with pre-pandemic depression.

### Depressive symptomatology levels in individuals with and without a history of prior depression and household food security status during the pandemic

**Fig. 2:**
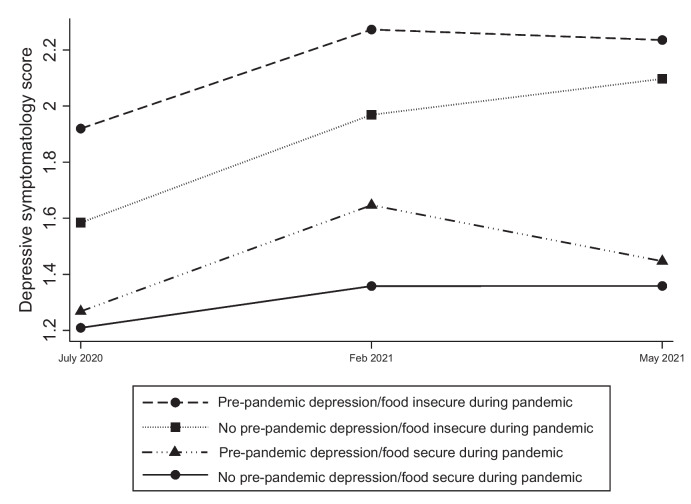
Depressive symptomatology levels in individuals with and without pre-pandemic depression and household food security during pandemic

The trends in depression levels in individuals with and without a history of prior depression in relation to household food security status are indicated in Fig. 2. Two important findings emerged, the first being that the highest depressive symptomatology was observed in those who had pre-pandemic depression and were food insecure during the pandemic. In contrast, the lowest was observed in those without pre-pandemic depression who were food secure during the pandemic, as anticipated. Second, in Wave 3 (Feb 2021) compared to Wave 2 (July 2020), depression levels rose by approximately equal magnitude in two groups: those without pre-pandemic depression but food insecure during pandemic (lincom results: adj β = 0.38, p < 0.01, ES = 0.21); and those with pre-pandemic depression but food secure during pandemic (lincom results: adj β = 0.38, p = 0.02, ES = 0.23). Thereafter, these groups diverged. While depression levels continued to rise in the former group in Wave 5, we observed a declining trend in the latter group, with the gap between them widening at Wave 5 (adj β = 0.79, p < 0.01, ES = 0.47) from Wave 3 (adj β = 0.63, p < 0.01, ES = 0.36).

Regarding other covariates in the regression model, we detected Black African participants [compared to White participants] (adj β = -0.73, p < 0.01), Asian/Indian participants [compared to White participants] (adj β = -0.58, p < 0.01), lack of comorbidity (adj β = -0.23, p < 0.01) and decreased household income during the pandemic (adj β = 0.20, p < 0.01) as being significantly associated with depression.

## Discussion

Our study observed the highest levels of depression among individuals with pre-pandemic depression who were food insecure during the pandemic; and the lowest among those without pre-pandemic depression and food secure during the pandemic. This highlights the vulnerability of the former group during the early stages of a crisis fuelled by the global pandemic/disaster. While depression levels rose in nearly equal magnitude during the early phases of the study in the two groups (pre-pandemic depression/food insecure group and no pre-pandemic depression/food secure group), differences emerged between them as the pandemic progressed. During the later phases, it appears that ongoing food insecurity constituted a more powerful driver of depression than a history of pre-pandemic depression.

The relationship between pre-existing mental illness and ongoing socio-economic hardships in the evolution of depression can be informed by both social drift theory and social causation theory (Dohrenwend et al., 1992; Hudson, 2005; Reiss, 2013). Social drift theory contends that individuals with mental illnesses tend to become socio-economically poorer over the course of their lives as a result of their health challenges, which in turn can further contribute to poor mental health (Lund et al., 2011). In contrast, social causation theory posits that mental illness is more likely to be experienced by individuals who are living in poverty due to the negative impact of adverse social and economic conditions (Dohrenwend et al., 1992; Hudson, 2005; Reiss, 2013). Our results appear to provide support for the latter mechanism, as ongoing food insecurity during the pandemic was associated with increasing depressive symptomatology, irrespective of whether or not there was a prior history of depression. Nevertheless, both social causation and social drift mechanisms were likely at play in this population, as a previous South African population study invoked a role for both in the complex interplay between poverty and depression (Lund and Cois, 2018).

Our study has several limitations, the first being that depression was self-reported, as data on the clinical diagnosis of depression was not available. While the CESD-10 was used in the SA-NIDS study, the SA-NIDS-CRAM utilized two-item version of the PHQ-2 due to shorter telephonic survey (Oyenubi et al., 2021) Although the PHQ-2 is a valid instrument for use in time-constrained settings (Oyenubi et al., 2021; Bhana et al., 2015), we opted not to use any cut-off, given its low sensitivity (Bhana et al., 2015). Second, although a food security measure was available in the SA-NIDS CRAM study, this was not available in the pre-pandemic 2017 SA-NIDS study. This means we could not explore the possible impact of long-term food insecurity predating the pandemic. The challenge of using a single-question screener for food security, possibly due to the need for a shorter telephonic survey in SA-NIDS-CRAM, will necessitate the use of a more comprehensive measure, such as Household Food Insecurity Access Scale (HFIAS) used in South Africa (Coates et al., 2007; Mlay et al., 2022) for future studies. Lastly, diagnostic data on COVID-19 was not available in the SA-NID-CRAM. Therefore, we cannot exclude the role of COVID, particularly in Wave 3, when the number of cases was high in South Africa (Mathieu et al., 2020).

Notwithstanding the limitations, our study has some important policy implications. The disaster, namely the COVID-19 pandemic, had a profound negative impact on depression, which was pronounced among the most socially vulnerable population (i.e. facing food insecurity), in a country with historically large mental health challenges and chronically high levels of poverty, even before the devastating pandemic.

## Recommendations

The COVID-19 global pandemic strained South African health systems in profound and manifold ways that disrupted access to health care for chronic conditions (Health Systems Trust, 2021) in a nation with a large mental health treatment gap (Docrat et al., 2019). Our study indicated that co-morbidity (i.e. which included HIV, TB, lung condition, heart condition or diabetes) also impacted depressive symptomatology. Given that context, there is an obvious need to address poor access to health services (Tomita et al., 2017) in addition to poverty, which contributes to food insecurity in South Africa (Mbajiorgu et al., 2022). Our study also highlights the importance of social protection, given the greater mental health challenge faced by food-insecure individuals. While the World Bank notes the mitigating role of South Africa’s social grants in coping with crises, disasters and associated economic shocks before and during a pandemic (Oosthuizen et al., 2021), poverty remains persistent in South Africa (Sulla et al., 2022). This research supports evidence that social protection/poverty alleviation can have multifaceted benefits for health and wellbeing, a necessary enhancement of resilience, especially given the rising frequency and intensity of crises.
